# Clinical trial update: treatment of metastatic brain tumors using MRgFUS

**DOI:** 10.1186/2050-5736-3-S1-O12

**Published:** 2015-06-30

**Authors:** Stephen Monteith, David Newell, Sandra Vermeulen, Charles Cobbs

**Affiliations:** 1 Swedish Neuroscience Institute, Seattle, WA, United States

## Background/introduction

Metastatic brain tumors are common lesions that are currently treated with multimodal therapy. Surgical resection, focused stereotactic radiosurgery, and whole brain radiation remain traditional therapies. It has been postulated that metastatic brain tumors could be treated effectively with MRgFUS using thermal ablation.

## Methods

Treatment of metastatic brain tumors using MRgFUS continues as an open clinical trial. As of the current time we have been unable to enroll patients in the current trial. There are several factors which have resulted in challenges in patient recruitment despite a large referral base.

## Results and conclusions

Many patients present with greater than 3 metastatic lesions and so are excluded from the clinical trial. Patients with metastatic malignancy to the brain will often have many tiny (in the order of a few mm on high resolution MRI) asymptomatic lesions which results in their exclusion in the current trial based on current criteria. Exclusion based on any prior hemorrhage in a metastatic lesion has also been raised as a concern. Many lesions may have tiny microhemorrhages on gradient echo imaging without clinically occult manifestation or clinical relevance. Metastatic tumors tend to present at the gray-white matter junction and not in the center of the brain which is more favorable for MRgFUS treatment. Exclusion of patients due to unfavorable tumor location based on the limited treatment envelope offered by current treatment systems remains a challenge. Experience with a large volume of screened patients has led to modifications suggested to current protocols in order to take in to consideration these findings.

